# Isolation and Annotation of Azira, a CT Cluster Phage That Infects Gordonia rubripertincta

**DOI:** 10.1128/mra.00347-23

**Published:** 2023-06-22

**Authors:** Coen E. E. McGarrah, Elijah D. Algarin-Martinez, Maria E. D. Cavasini, Victoria Correa, Don F. Danielson, Wynter R. Dean, Joseph L. French, Naleena N. Gaskin, Urvi Jain, Jeanette Janvier, Breanna M. Macumber, Francesca Kristal Martini, Sydney G. Mazzei, Jennifer M. Mujica, Oslow Odegaard, Corissa Quaterman, Taylor M. Rand, Nina S. Seidensticker, Tyler Serrano, Anna Soltys, Maxwell D. Ungrey, Richard S. Pollenz

**Affiliations:** a Department of Molecular Biosciences, University of South Florida, Tampa, Florida, USA; Loyola University Chicago

## Abstract

Azira is a CT cluster actinobacteriophage that infects Gordonia rubripertincta NRRL B-16540. The genome contains 67 predicted protein coding genes, of which 31 have a putative function. Azira has a lysis cassette encoding two endolysins and three transmembrane proteins. Azira contains four genes predicted to encode enzymes involved in thymine synthesis.

## ANNOUNCEMENT

Bacteriophages are one of the most common organisms on Earth (1). The SEA PHAGES program has annotated approximately 4,400 actinobacteriophages (2, 3), and the discovery and characterization of new phage genomes have been important in furthering the understanding of phage genomics and evolution.

Azira was isolated from a moist soil sample from Tampa, Florida (28.071111 N, 82.413056 W), by shaking at 250 rpm for 2 h in peptone-yeast calcium media (PYCa) followed by sterile filtration. Gordonia rubripertincta NRRL B-16540 was infected with sterile soil lysates or pure phage and plated onto PYCa agar at 30°C. Genomic DNA was isolated after three rounds of plaque purification using the Wizard DNA cleanup kit (A7280; Promega). Genomic DNA was used to create sequencing libraries with the New England BioLabs (NEB) ultra II library kit with v3 reagents. Sequencing was performed by the Pittsburgh Bacteriophage Institute, and the library was run on an Illumina MiSeq instrument, yielding 242,293 paired-end 150-base pair reads yielding a 777-fold average coverage. Raw reads were assembled with Newbler (v2.9) (4), yielding a single phage contig. The results were checked for completeness, accuracy, and genome termini using Consed (5). Default parameters were used for all software unless otherwise specified. Azira is 45,336 bp with 3′ sticky overhangs (CGGTAGGCAT) and was bioinformatically linearized such that base 1 was assigned in accordance with other *Gordonia* phages (6). Azira was autoannotated using DNA master (v5.23.6) (7), and the genes were then manually validated for correct starts and functional calls. GeneMark (v2.5) (8) and Glimmer (v3.02) (9) were utilized to assess start sites and coding potential, and Starterator (v1.2) (3) was used to summarize the starts across each family of phage genes. Evidence to support a gene product function was collected using HHpred (v3.2) (10), NCBI BLAST (11), and the Conserved Domain Database (12). Putative transmembrane (TM) domains were identified using Deep TMHMM and (13) TOPCONS (14). tRNAscan-SE (v2.0) (15) and ARAGORN (v1.2.41) (16) were utilized to identify putative tRNAs and transfer-messenger RNAs (tmRNAs). The data for Azira were archived in Phamerator (17) and the Actinobacteriophage Database at PhagesDB.org (2) (https://phagesdb.org/phages/Azira/).

**FIG 1:**
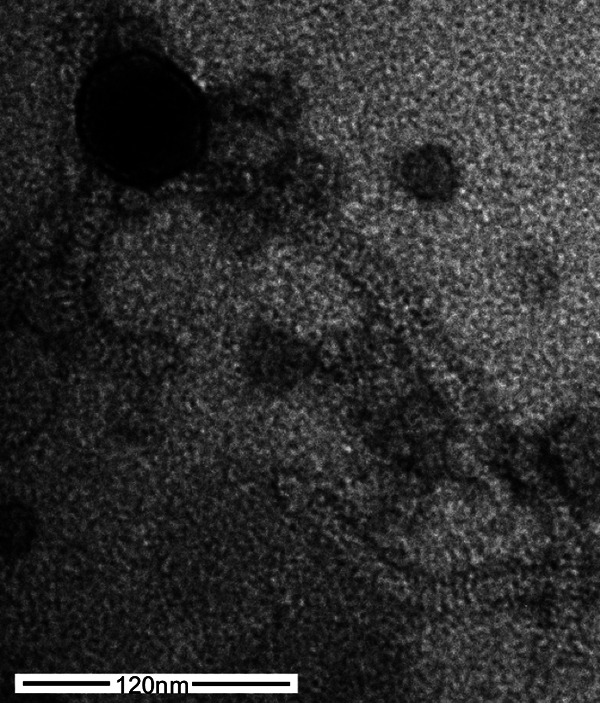
Transmission electron micrograph of *Gordonia* phage Azira (https://phagesdb.org/phages/Azira/). Phage lysates were negatively stained with 1% uranyl acetate. White scale bar = 120 nm.

Negative-staining transmission electron microscopy shows that Azira has a 289-nm tail and an icosahedral capsid of ~50 nm (Fig. 1). Azira is one of 40 annotated CT cluster phages and contains 67 predicted protein-coding genes, of which 31 have a predicted function. All CT cluster phages have endolysin genes that encode proteins grouped to the same family (3). However, the CT phages have four variations in the linear organization and topology of the encoded TM proteins thought to be involved in host lysis (18) (Table 1). Azira has the most common organization (25 phages) with three encoded TM proteins with 2, 4, and 1 TM domain. Azira also contains the following four genes in an operonic-like organization that encode enzymes predicted to be involved in thymine synthesis: deoxycytidylate deaminase (gp41), thymidylate synthase (gp43), dUTPas (gp46), and thymidylate kinase (gp47). An analysis of these genes across all phages in the PhagesDB database (3) showed that the CT cluster phages are the only ones to have all four.

**TABLE 1:** Genome organization of genes encoding putative transmembrane proteins in the lysis cassette of CT cluster phages^*a*^

CT cluster phage	GenBank accession no.	Host strain^*b*^	Genome (bp)	First TM protein	Second TM protein	Third TM protein	Fourth TM protein
Agatha	MK875795	GT	46,543	gp22 (2)	gp23 (4)	gp24 (1*)	
AikoCarson	MZ322013	GT	45,241	gp21 (2)	gp22 (4)	gp23 (1*)	
Amok	OL829977	GR	45,278	gp21 (2)	gp22 (4)	gp23 (1*)	
AndPeggy	MN586036	GR	44,609	gp21 (2)	gp22 (4)	gp23 (1*)	
Axym	MN444871	GT	46,473	gp22 (2)	gp23 (4)	gp24 (1*)	
Azira	OQ709211	GR	45,336	gp24 (2)	gp25 (4)	gp26 (1*)	
BigChungus	MT776810	GR	47,166	gp21 (4)	gp22 (2)	gp23 (2)	
Biskit	ON970599	GT	45,847	gp23 (2)	gp24 (4)	gp25 (1*)	
Burnsey	MT889378	GT	46,185	gp22 (2)	gp23 (4)	gp24 (1*)	
Button	ON970621	GR	46,090	gp23 (2)	gp24 (2)	gp25 (2)	
Buttrmlkdreams	MT776809	GT	45,999	gp22 (2)	gp23 (4)	gp24 (1*)	
CherryonLim	MN284906	GR	48,948	gp23 (4)	gp24 (2)	gp25 (2)	
Cleo	MN586057	GR	44,795	gp22 (2)	gp23 (4)	gp24 (1*)	
Cozz	KU998239	GT	46,600	gp222 (2)	gp23 (4)	gp24 (1*)	
Dre3	MW507135	GR	45,810	gp22 (2)	gp23 (4)	gp24 (1*)	
Emalyn	KU963260	GT	43,982	gp21 (2)	gp22 (4)	gp23 (1*)	
Feastonyeet	MT776808	GR	47,166	gp21 (4)	gp22 (2)	gp23 (1)	
Gibbous	MN310549	GR	45,810	gp222 (2)	gp23 (4)	gp24 (1*)	
GiKK	OL455888	GR	47,537	gp25 (2)	gp26 (2)	gp27 (1*)	
GTE2	HQ403646	GT	45,540	Not called	gp20 (4)	gp21 (1*)	
Hexbug	ON970609	GR	47,190	gp24 (2)	gp25 (2)	gp26 (1*)	
Lauer	MN586015	GR	48,123	gp21 (4)	gp22 (2)	gp23 (2)	
Margaret	MH271302	GT	46,950	gp25 (2)	gp26 (2)	gp27 (2)	
Mayweather	MN062716	GR	48,382	gp23 (4)	gp24 (2)	gp25 (2)	
MScarn	MZ150793	GT	45,677	gp24 (2)	gp25 (4)	gp26 (1*)	
Nina	MK279900	GT	46,702	gp23 (2)	gp24 (4)	gp25 (1*)	
Orla	MT889367	GR	47,354	gp24 (2)	gp25 (2)	gp26 (2)	
Pons	OK040785	GR	47,982	gp222 (4)	gp23 (2)	gp24 (2)	
Quasar	MN444878	GT	46,960	gp22 (2)	gp23 (4)	gp24 (1*)	
SheckWes	MK967385	GR	47,618	gp21 (4)	gp22 (2)	gp23 (2)	gp24 (1*)
SketchMex	MH450132	GT	45,847	gp21 (2)	gp22 (4)	gp23 (1*)	
SteamedHams	MN234215	GR	44,571	gp24 (2)	gp25 (4)	gp26 (1*)	
SummitAcademy	OP297531	GR	47,328	gp21 (4)	gp22 (2)	gp23 (2)	gp24 (1*)
Survivors	ON970576	GR	45,436	gp24 (2)	gp25 (4)	gp26 (1*)	
SweatNTears	MK967383	GT	45,917	gp24 (2)	gp25 (4)	gp26 (1*)	
Tolls	MW862988	GT	44,786	gp24 (2)	gp25 (4)	gp26 (1*)	
Troje	MG770215	GT	45,909	gp22 (2)	gp23 (4)	gp24 (1*)	
Vine	MZ622167	GR	48,092	gp23 (4)	gp24 (2)	gp25 (2)	
Yakult	MK875791	GT	47,197	gp23 (2)	gp24 (2)	gp25 (2)	
Yarn	MN586049	GR	44,619	gp21 (2)	gp22 (4)	gp23 (1*)	

a The four different organizations of the putative transmembrane domain (TM) encoding genes in the lysis cassette of CT cluster phages. CT cluster phages are listed alphabetically with GenBank accession nos. Azira is highlighted for reference. TMs were identified in the encoded proteins using TOPCONS (14) and Deep TMHMM (13). The gene product no. is shown, and the predicted no. of TM domains are presented for each gene in parenthesis. No. with an asterisk predict a single TM domain that may serve as a signal sequence.

b GR, Gordonia rubripertincta NRRL B-16540; GT, Gordonia terrae 3612.

### Data availability.

This whole-genome shotgun project has been deposited in DDB/ENA/GenBank under the accession no. OQ709211 and SRX19690841. The version described in this paper is the first version.
